# Development and Validation of Nurses’ Well-Being and Caring Nurse–Patient Interaction Model: A Psychometric Study

**DOI:** 10.3390/ijerph18157750

**Published:** 2021-07-21

**Authors:** Hui-Chun Chung, Yueh-Chih Chen, Shu-Chuan Chang, Wen-Lin Hsu, Tsung-Cheng Hsieh

**Affiliations:** 1 Department of Nursing, Hualien Tzu Chi Hospital, Hualien 970, Taiwan; a0970332633@yahoo.com.tw; 2 Department of Nursing, Tzu Chi University, Hualien 970, Taiwan; scchang@mail.tcu.edu.tw; 3 Department of Nursing, National Taiwan University, Taipei 100, Taiwan; ychichen@ntu.edu.tw; 4 Nursing Committee, Hualien Tzu Chi Hospital, Hualien 970, Taiwan; 5 Hualien Tzu Chi Hospital, Hualien 970, Taiwan; hwl@tzuchi.com.tw; 6 Medical Department, Tzu Chi University, Hualien 970, Taiwan; 7 Institute of Medical Sciences, Tzu Chi University, Hualien 970, Taiwan; 8 Doctoral Degree Program in Translational Medicine, Tzu Chi University and Academia Sinica, Hualien 97004, Taiwan

**Keywords:** care nurse–patient interaction, health-promoting, well-being, work environment satisfaction, structural equation modeling

## Abstract

Nurses’ care nurse–patient interaction (CNPI) competence is critical for improving nursing care quality. The focus is the psychological quality of nurses, which may be derived from their sense of well-being. The purpose of this study was to develop a conceptual model of nurses’ well-being and their CNPI competence. A cross-sectional survey was conducted with a total of 212 valid questionnaires obtained from a medical center. Structural equation modeling analysis was performed to validate the conceptual model. The results demonstrated the positive correlation between two constructs of nurses’ well-being (“contentment” and “joyfulness”) and CNPI competence. A positive correlation between nurses’ CNPI competence and their health-promoting lifestyle and work environment satisfaction was supported. Among the constructs of CNPI competence, “respect patients’ life experience” was the most correlated with their well-being, health-promoting lifestyle, and work environment satisfaction. The constructs of these three scales, which showed a middle correlation with CNPI competence, are psychological constructs rather than material constructs. When nurses have a greater sense of well-being, a positive attitude towards life, and feel supported and respected in their work environment, their CNPI competence increases. The findings of this study provide important insights and can serve as empirical evidence for nursing managers to enhance nurses’ CNPI competence.

## 1. Introduction

Nurse–patient interaction refers to nurses’ ability to understand their patients’ life experiences, the influence of the diseases on patients, and the health expectations of patients through dialogue between nurses and patients. A nurse–patient relationship can be formed, and more suitable care can be provided to patients with this understanding [1]. When nurses express care and learn about their patients’ life experiences, they are able to reflect and improve their professional skills. Patients can also gain a sense of security and obtain therapeutic benefits during these interactions with nurses. Building this trusting relationship with patients makes them more willing to receive the provided care during complicated situations and feel more satisfied with the quality of nursing care. The positive feedback from patients also gives nurses encouragement in their work while leaving them feeling joyful in their professional capacity. As such, experts and scholars strongly recommend that nurse–patient interaction and care be considered an important professional nursing competence [2,3].

Chung et al. [4] developed the Chinese version of the scale for Caring Nurse–Patient Interaction (CNPI) competence based on the CNPI scale from Cossette et al. [5,6,7]. Their results showed the CNPI competence for Chinese clinical nurses consisted of four constructs: (1) help patients feel comfortable, (2) afford care according to patient needs, (3) respect patients’ life experiences, and (4) help patients achieve their health expectations. These results suggest that nurse–patient interactive care is centered on patients from the perspective of Chinese clinical nurses. The results further show that nurses attained the highest scores for the construct “provide care according to patient needs” while they attained the lowest scores for the construct “help patients achieve their health expectations”. This implies that the nurses have confidence in their professional skills. However, the nurses need to improve their competence in considering the impact of illness from the patients’ point of view. Nurses need to focus more on understanding patients’ life experiences and patients’ own health expectations. By fulfilling these requirements, nurses can jointly develop care plans with patients and provide individual and personalized nursing care.

“Well-being” refers to a person’s perceptions of influential factors in the surrounding environment. Dodge et al. [8] defined “well-being” as the balance point between an individual’s resource pool and the challenges they face. The question of what “well-being” is has prompted multiple definitions and studies of this term in Eastern and Western cultures. Studies conducted in Western countries have indicated that “well-being” refers to the extent to which a person favors his or her living condition and the physical, psychological, and social comforts and happiness he or she derives from everyday life. Well-being enables people to gain maximum satisfaction in life and is a multidimensional concept that comprises various factors. One study revealed that well-being involves the following constructs: (1) positive affect, (2) negative affect, and (3) life satisfaction [9]. Lu [10] proposes the Eastern concept of well-being, asserting that well-being is a type of continuous positive force and that it refers to overall satisfaction in life. Lu categorized the following nine major dimensions for measuring the well-being of Chinese people: satisfaction with self-esteem, harmonious interpersonal relationships with family and friends, the pursuit of money, work achievement, optimistic attitude toward life, living a better life than others, self-control and fulfillment of ideals, short-term happiness, and health needs [11].

Chung et al. [12] developed the Chinese version measurement scales and conceptual structural model for nurses’ well-being, health-promoting lifestyles, and work environment satisfaction. Their results showed that the well-being of Chinese nurses consists of two constructs: “contentment” and “joyfulness”. “Contentment” represents the level of satisfaction with lifestyle and life situation. “Joyfulness” represents the level of optimism about the present and the future. Chung’s model further demonstrated the positive correlation between nurses’ well-being, health-promoting lifestyles, and work environment satisfaction. Among the three constructs (activities, attitude, and companionship) of a health-promoting lifestyle and the five constructs of work environment satisfaction (benefits, support, respect, security, and facilities), the aspects of attitude, companionship, support, and respect were all positive mental attitudes that supported the sense of well-being. This implies that for clinical nurses, their source of well-being is based primarily on the psychological and positive feedback in their life and work environment.

CNPI competence emphasizes that nurses understand the patient’s life experience and feelings while ill, as well as the expectation of their own health through positive interaction with nurses. While nursing staff are caring for patients, it is equally important that they have stable psychology for interacting with patients. Is a nurse’s psychological quality derived from their sense of well-being composed of contentment and joyfulness? At present, the relationship between nurses’ well-being and their CNPI competence has yet to be established. The purpose of this study was to develop the conceptual structural model between nurses’ well-being and their CNPI competence to evaluate the following two hypotheses:

**Hypothesis**  **H1.**  *Contentment was positively correlated with CNPI competence*.

**Hypothesis**  **H2.**  *Joyfulness was positively correlated with CNPI competence*.

**Figure 1:**
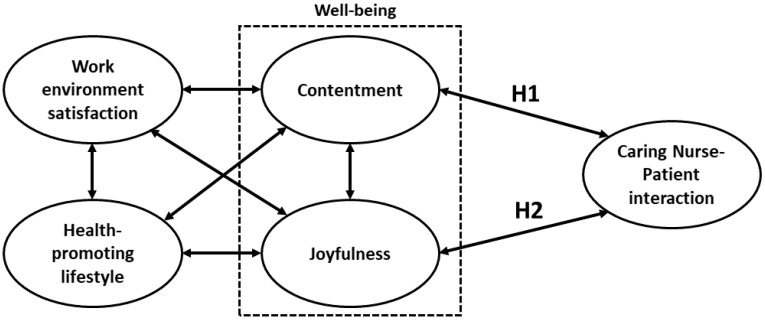
Hypothetical model of nurses’ well-being vs. CNPI competence. The part on the left of H_1_ and H_2_ is the WHS model proposed by Chung et al. [12].

In addition to these two hypotheses, the correlation of nurses’ CNPI competence with their health-promoting lifestyle and work environment satisfaction were also evaluated. Based on the proposed hypotheses, this study submitted the framework in Figure 1 to validate the corresponding structural models.

## 2. Materials and Methods

### 2.1. Design and Participants

**Figure 2:**
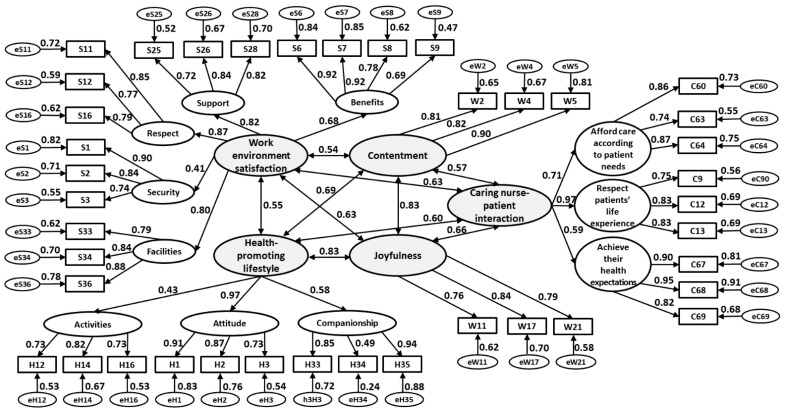
The final structural model of well-being versus CNPI competence.

This study was conducted based on a cross-sectional design for developing the structural model of the relationship between nurses’ well-being and their CNPI competence. The stratified random sampling method stratified by department was employed to enroll the study participants from a medical center in Taiwan. Bentler and Chou [13] suggested a ratio of 5 observations per observed variable as sufficient for structural estimating equation (SEM) analysis. Thompson [14] recommends the minimum sample size requirement for SEM analysis at 200. There were a total of 40 scale items to be measured in the model (Figure 2), which resulted in the sample size of 200 (40 × 5) required. With a projected 80% return rate, a total of 250 registered nurses were invited to attend the study, yielding at least 200 valid questionnaires. The purpose and procedures of the study were explained to the nurses by the researcher. The nurses who chose to participate in the study were invited to a meeting room at an arranged time. Participants were given informed consent forms and apprised of their right to withdraw from the study at any time for any reason. The questionnaire was given to the participants and comprised two parts, including (1) participant information: age, marital status, education level, and tenure and (2) study scales: the Nursing Health and Job Satisfaction (NHJS) Scale [12] and the Chinese Comfort, Afford, Respect, and Expect (CARE) scale [4] for collecting the data of nurses’ well-being and CNPI competence. Questionnaires were checked and confirmed for completeness at the end of the session. This resulted in a total of 212 valid questionnaires collected, with an 84.8% return rate that met the sample size requirement.

### 2.2. Measures and Variables

The NHJS Scale and the Chinese CARE scale developed by Chung et al. [4,12] were employed for collecting the data in the present study. Chung et al. [12] proposed the NHIS score for assessing and validating the relationship of nurses’ well-being, health-promoting lifestyles, and work environment satisfaction. The Cronbach’s α of the three subscales ranged from 0.83 to 0.91, showing good reliability of the scale. The total variances explained by the three subscales ranged from 92.6% to 95.1%, indicating the excellent ability and validity of the NHJS scale for evaluating nurses’ well-being, work environment satisfaction, and health-promoting lifestyles. The Chinese CARE scale was adapted for assessing the CNPI competence of nurses [4]. Cronbach’s α of 0.91 and the total variances explained by the scale of 95.9% showed favorable reliability and excellent ability and validity for evaluating nurses’ CNPI competence of the CARE scale.

Both scales are 5-point Likert-style self-assessment questionnaires with the score ranging from 1 to 5 for each item. The NHJS scale compromises three subscales, including the well-being scale with 6 items and 2 constructs (“contentment” and “joyfulness”), the work environment subscale with 16 items and 5 constructs (“benefit”, “support”, “respect”, “security”, and “facilities”), and the health-promoting lifestyle subscale with 9 items and 3 constructs (“activities”, “attitude”, and “companionship”) for assessing hospital nurses’ well-being, work environment satisfaction, and health-promoting lifestyles. The score for each of the two constructs for the well-being scale ranged from 3 to 15. The scores ranged from 16 to 80 for the work environment subscale and 9 to 45 for the health-promoting subscale. Higher scores in the well-being subscale, work environment subscale, and health-promoting subscale represent a higher level of well-being, high satisfaction, and a greater tendency toward leading health-promoting lifestyles. The CARE scale comprises 12 items with 4 constructs, including “help patients feel comfortable”, “afford care according to patient”, “respect patients’ life experiences”, and “help patients achieve their health expectations”. The range of scores was from 12 to 60. A higher score represents higher CNPI competence.

### 2.3. Data Analysis

Analysis was performed using AMOS for Windows, Version 21.0. Armonk, NY, USA: IBM Corp. Structural equation modeling (SEM) was used to validate the conceptual model in Figure 1. The path coefficients (PC) of the variables in the structural model were estimated for evaluating the relationship between CNPI competence and nurses’ well-being, health-promoting lifestyles, and work environment satisfaction. In order to validate the goodness of fit in the model (Figure 1) and the strength of association of the model, χ^2^/df, the goodness-of-fit index (GFI), root mean square error of approximation (RMSEA), parsimonious normed fit index (PNFI), and the comparative fit index (CFI) were calculated. The corresponding criteria were χ^2^/df < 3, GFI > 0.8, RMSEA < 0.08, PNFI > 0.5, and CFI > 0.9, respectively [15]. The modification index (MI) obtained by SEM was considered as the basis for adjusting the path relationship of the variables if the corresponding MI ≥ 3.84. A *p* value < 0.05 was considered statistically significant.

## 3. Results

### 3.1. Participant Characteristics and Scale Score

Characteristics of the participants are summarized in Table 1. Among the total 212 participant nurses, the mean age and tenure were 29 and 7.5 years, respectively. In total, 62.3% of them were younger than 30 years old, while 72.6% of them had worked as nurses for more than 2 years. Only 28.8% were married, and 61.8% held a bachelor’s degree or higher education. A total of 33.4% of the nurses worked in emergency and critical units, and most (39.6%) were in level III (N2). Summary statistics of the scale scores and their reliability coefficients are summarized in Table 2. Cronbach’s α ranged from 0.84 to 0.91, showing the favorable reliability of the CARE scale and NHJS scale based on the collected data in the present study. A mean score of 45.0 for the CARE score showed the middle CNPI competence for the participating nurses. Mean scores of the two constructs of the well-being subscale (“contentment”: 10.0, “joyfulness”: 10.6), work environment satisfaction (53.8), and health-promoting lifestyle (27.6) showed the low to very low level of sense of well-being, less satisfaction with the work environment, and poor health-promoting lifestyle.

### 3.2. Findings of SEM Analysis

The final structural model of well-being versus CNPI competence is presented in Figure 2 via SEM analysis. The CNPI construct “help patients feel comfortable” was removed from the model due to unreasonable standardized factor loading of 1.04. The calculated indices of goodness of fit of model (χ^2^/df = 1.53, GFI = 0.80, RMSEA = 0.05, PNFI = 0.76, and CFI = 0.93) showed the final model fit the data well. The hypotheses H1 and H2 are supported by the correlation between the two constructs of nurses’ well-being and CNPI competence (Table 3). Both contentment (PC = 0.57) and joyfulness (PC = 0.66) were significantly and positively correlated with nurses’ CNPI competence (PC = 0.66). Furthermore, the results of path analysis in Figure 2 showed not only well-being but also nurses’ work environment satisfaction (PC = 0.63, Z = 4.96, *p* < 0.001) and health-promoting lifestyles (PC = 0.60, Z = 4.46, *p* < 0.001) had a significant positive correlation relationship with CNPI competence.

The correlation coefficients of the constructs of well-being, work environment satisfaction, and health-promoting lifestyle with CNPI competence are enumerated in Table 4 for further clarification of their relationship.

Among the three constructs of CNPI competence, “afford care according to patient needs” showed a middle correlation (i.e., *r* ≥ 0.4) with “joyfulness” (*r* = 0.424) of well-being and “attitude” (*r* = 0.407) of health-promoting lifestyle. “Respect patients’ life experience” showed a middle correlation with both “contentment” (*r* = 0.477) and “joyfulness” (*r* = 0.558) of well-being, “support” (*r* = 0.472), and “respect” (*r* = 0.474) of work environment satisfaction, and “attitude” (*r* = 0.516) of health-promoting lifestyle. For the construct “help patients achieve their health expectations”, it showed a middle correlation only with “contentment” (*r* = 0.403) of well-being. By contrast, “security” (*r* = 0.075–0.207) of work environment satisfaction, “activities” (*r* = 0.171–0.182), and “companionship” (*r* = 0.191–0.251) of health-promoting lifestyle showed low correlation with all three constructs of CNPI competence. These results show that “respect patients’ life experience” of CNPI competence was the main construct establishing the relationship of nurses’ CNPI competence with well-being, work environment satisfaction, and health-promoting lifestyle. The constructs of these three scales showing middle correlation with CNPI competence are psychological constructs (“contentment”, “joyfulness”, “support”, “respect”, and “attitude”) rather than material (“benefits”, “security”, and “activities”).

## 4. Discussion

In this study, the structural model of well-being versus CNPI competence for Chinese clinical nurses was developed and validated. The results of the model validation demonstrated that the developed model fit the data well. The validated model showed that nurses’ well-being, work environment satisfaction, and health-promoting lifestyle were positively correlated to their CNPI competence. Among the three constructs of nurses’ CNPI competence, “respect patients’ life experience” was most correlated with their well-being, work environment satisfaction, and health-promoting lifestyles.

Louise et al. [16], after reviewing 46 studies, indicated that poor well-being and moderate to high levels of burnout for clinical nurses were associated with poor patient safety outcomes due to medical errors. Paula and Martin [17] also confirmed the importance of maintaining psychosocial well-being in the workplace for palliative care nurses to be able to undertake their work in the best possible way. Arrogante and Perez-Garcia [9] studied the relevance of nursing work environments to the well-being of nurse staff members. They revealed that subjective well-being, including satisfaction with life, positive affect, and negative affect, is influenced directly by personality and resilience. Moreover, the results revealed that subjective well-being was associated with greater resilience and less neuroticism in the nursing staff. In the current study, both constructs (“contentment” and “joyfulness”) of nurses’ well-being were positively correlated with their CNPI competence (PC = 0.57, 0.66). When nurses’ well-being increases, their resilience also increases. This enables them to have a more stable psychological quality with an improved nursing ability to understand and care for patients’ needs. From the correlation analysis between the constructs of nurses’ well-being and CNPI competence, “contentment” and “joyfulness” of well-being and “respect patients’ life experience” of CNPI competence showed the strongest correlation (*r* = 0.477, 0.558). This implies that when the nursing staff feel satisfied and joyful from their efforts in caring for patients, they are also better able to aid patients when their health is threatened. They can do this and select the most appropriate nursing process based on the patient’s life experience while respecting the patient’s health cognition and expectations.

Pender et al. [18] proposed that a health-promoting lifestyle is a type of approach behavior in their theory of the health promotion model. It refers to behaviors that maintain or increase well-being and achieve self-realization and personal accomplishments. A literature review by Pérez-Francisco et al. [19] showed that nurses’ health suffered due to high pressure of care, which contributed to the impairment of the quality of care and patient safety. This indicates the importance of nurses’ mental health on the quality of patient care. In the current study, nurses’ health-promoting lifestyles were positively correlated with their CNPI competence (PC = 0.60). Further exploration into the relationship between the constructs of nurses’ health-promoting lifestyles and CNPI competence, “attitude” of health-promoting lifestyles and “respect patients’ life experience” of CNPI competence showed the highest correlation (*r* = 0.516). These results indicate that when nurses have a positive attitude, they are able to understand life experiences that affect patients, respect their opinions, make joint decisions, and carry out patient-centered care.

Recent literature demonstrates the positive relationship between job satisfaction and caring behaviors for nurses [20]. Improvement of nursing work environments and supporting nurses can improve nursing outcomes and promote quality care [21]. Nurse–physician relations, nurse managers’ leadership ability, and the support of nurses are the most critical characteristics of a work environment [22]. The current study shows the positive correlation between nurses’ work environment satisfaction and their CNPI competence (PC = 0.63). From the correlation analysis of the current study between the constructs of nurses’ work environment satisfaction and CNPI competence, “respect” and “support” of work environment and “respect patients’ life experience” of CNPI competence showed the highest correlation (*r* = 0.472, 0.474) among constructs. This implies that when nurses feel respected in the working environment, their self-affirmation improves, and they become more self-confident with their caring skills. With support from supervisors and colleagues, they are able to transform the sense of being respected into respecting the life experience of the patient. At the same time, they are more willing to support and respect the feelings of the patient, thereby enhancing the caring ability of the patients.

Among the three constructs of nurses’ CNPI competence, “respect patients’ life experience” was the one that was most correlated with their well-being, work environment satisfaction, and health-promoting lifestyles. The constructs of three scales that showed middle correlation with CNPI competence are all psychological constructs (“contentment”, “joyfulness”, “support”, “respect”, and “attitude”) rather than material constructs (“benefits”, “security”, and “activities”) (Table 4). According to the scale items for measuring “respect patients’ life experience” in the CARE scale [4], “respect patients’ life experience” represents the ability to respect patients’ thoughts, guide them to positively face their health conditions, and help them seek motivation for promoting their health. However, nurses may also suffer as a result of patients’ negative emotions during their interaction with patients if they have no strong psychological quality. Our study demonstrates that nurses’ psychology for caring for patients can be enhanced via the improvement of the psychological elements of their well-being, work environment satisfaction, and health-promoting lifestyles.

The positive correlation between the CNPI competence and nurses’ well-being, health-promoting lifestyles, and work environment satisfaction forms a virtuous cycle. Increases in the sense of nurses’ well-being, health-promoting lifestyles, and satisfaction within their work environment can enhance nurse’s CNPI competence. As nurses’ CNPI competence improves and positive feedback from the patients increases, they feel encouraged and gain an improved sense of well-being. This also encourages them to change their health-promoting lifestyle and feel more satisfied with the work environment.

This study is the first to present a conceptual model for assessing the relationship between nurses’ well-being and CNPI competence. The insights gained from this study may be employed by nursing management teams to improve nurses’ CNPI competence. This study was limited in its scope as it was conducted in only one hospital. There may also be cultural differences that affect the interpretations of the data due to social norms. The capacity to generalize the results on a more global scale remains limited due to the inability to conduct this research in multiple countries and hospitals. To improve the generalizability of the study results and investigate possible cultural differences, a study including other countries and more hospitals can be further considered.

## 5. Conclusions

The validated model in this study demonstrates a positive correlation and a virtuous cycle among nurses’ CNPI competence, health-promoting lifestyles, well-being, and work environment satisfaction. “Respect patients’ life experience” was the construct of nurses’ CNPI competence that most correlated with their well-being, work environment satisfaction, and health-promoting lifestyles. When nurses had a greater sense of well-being, a positive attitude towards life, and felt supported and respected in their work environment, their CNPI competence increased. The findings of this study provide important insights and serve as an empirical basis for hospital management to better understand the factors that affect nursing care. Establishing appropriate management programs to enhance nurses’ CNPI competence and improve the quality of care is a mutually beneficial goal.

## Figures and Tables

**Table 1:** Demographic characteristics of the study participants.

Variables	Mean ± SD.	N (%)
Age (years)	29.0 ± 7.2	
<30 years		132 (62.3)
>=30 years		80 (37.7)
Marital status		
Married		61 (28.8)
Others		151 (71.2)
Education		
College		111 (38.2)
Bachelor or above		185 (61.8)
Tenure (years)	7.5 ± 7.1	
<=2 years		58 (28.4)
>2 years		154 (72.6)
Affiliated unit		
Emergency and critical unit		71 (33.4)
Others		141 (66.6)
Clinical ladder		
N		39 (18.4)
N1		42 (19.8)
N2		84 (39.6)
N3		35 (16.5)
N4		12 (5.7)

SD.: standard deviation.

**Table 2:** Reliability coefficients and summary statistics of scale scores.

Scale	Cronbach’s α	Mean ± SD.	Med. (Q1, Q3)	Min., Max.
CARE scale	0.91	45.0 ± 5.8	45.0 (41.0, 48.0)	28.0, 60.0
NHJS scale				
Well-being subscale: Contentment	0.87	10.0 ± 2.4	10.0 (9.0, 12.0)	3.0, 15.0
Well-being subscale: Joyfulness	0.84	10.6 ± 2.1	11.0 (9.0, 12.0)	3.0, 15.0
Work environment satisfaction subscale	0.91	53.8 ± 9.2	54.0 (48.0, 61.8)	25.0, 79.0
Health-promoting lifestyle subscale	0.84	27.6 ± 5.9	27.0 (24.0, 32.0)	14.0, 43.0

SD.: standard deviation, Med.: median, Q1: 25th percentile, Q3: 75th percentile, Min.: minimum, Max.: maximum.

**Table 3:** Path coefficients of the structural model corresponded to hypotheses H_1_ And H_2_.

Hypothesis		PC ^1^	Z	*p*-Value
H_1_	Contentment was positively correlated with CNPI competence	0.57	5.30	*p* < 0.001
H_2_	Joyfulness was positively correlated with CNPI competence	0.66	5.31	*p* < 0.001

^1^ PC: path coefficient.

**Table 4:** Correlation coefficients of the constructs of work environment satisfaction, health-promoting lifestyle, and well-being with CNPI competence.

Subscale of NJHS Scale	Construct	CNPI Competence					
		Afford Care According to Patient Needs		Respect Patients’ Life Experience		Help Patients Achieve Their Health Expectations	
		*r*	*p*-Value	*r*	*p*-Value	*r*	*p*-Value
Well-being	Contentment	0.377	<0.001	0.477	<0.001	0.403	<0.001
	Joyfulness	0.424	<0.001	0.558	<0.001	0.332	<0.001
Work environment satisfaction	Benefits	0.192	0.005	0.350	<0.001	0.285	<0.001
	Support	0.342	<0.001	0.472	<0.001	0.290	<0.001
	Respect	0.375	<0.001	0.474	<0.001	0.325	<0.001
	Security	0.075	0.276	0.207	0.002	0.184	0.007
	Facilities	0.324	<0.001	0.396	<0.001	0.265	<0.001
Health-promoting lifestyle	Activities	0.177	0.010	0.182	0.008	0.171	0.012
	Attitude	0.407	<0.001	0.516	<0.001	0.383	<0.001
	Companionship	0.209	<0.001	0.251	<0.001	0.191	<0.005

## Data Availability

The data that support the findings of this study are available on request from the corresponding author, T.-C.H. The data are not publicly available due to their containing information that could compromise the privacy of research participants.
